# Yellow Laser Stimulation at GV2 Acupoint Mitigates Apoptosis, Oxidative Stress, Inflammation, and Motor Deficit in Spinal Cord Injury Rats

**DOI:** 10.1155/2018/5407052

**Published:** 2018-10-08

**Authors:** Parichat On-ong-arj, Jintanaporn Wattanathorn, Supaporn Muchimapura, Wipawee Thukham-mee

**Affiliations:** ^1^Department of Physiology (Neuroscience Program), Faculty of Medicine, Khon Kaen University, Khon Kaen, 40002, Thailand; ^2^Department of Physiology, Faculty of Medicine, Khon Kaen University, Khon Kaen, 40002, Thailand; ^3^Integrative Complementary Alternative Medicine Research and Development Center, Khon Kaen University, 40002, Thailand

## Abstract

Currently, the suppression of oxidative stress and inflammation is considered as the treatment targets of spinal cord injury due to their roles on the hindrance of recovery process. Since laser acupuncture decreased oxidative stress and enhanced the survival of neurons from oxidative stress damage and GV2 stimulation was selected as one stimulated acupoint in order to enhance the recovery of spinal cord injury, we hypothesized that laser acupuncture at GV2 should enhance the recovery of spinal cord injury. To test this hypothesis, male Wistar rats were induced spinal cord injury at T10 level and they were exposed to a 10 minute-stimulation at GV2 by yellow laser. Laser acupuncture was performed at 0.25 and 1, 2, 6, and 12 hours after spinal cord injury. Then, the stimulation was performed once daily for 7 days. Locomotor assessment was carried out on days 3 and 7 after injury. At the end of study period, the densities of polymorphonuclear of leukocyte, Bax, Caspase-3, Bcl-2, and BDNF positive stained cells in ventral horn of spinal cord were determined. Cyclooxygenase-2 (COX-2), interleukin-6 (IL-6), and oxidative stress status was also assessed. The results showed that laser acupuncture at GV2 increased BBB score, gross motor score, and densities of Bcl-2 and BDNF positive stained cells but decreased density with polymorphonuclear leukocyte, the densities of Bax and Caspase-3 positive stained cells, COX-2 level, and oxidative stress status in ventral horn of the lesion spinal cord. The reduction of serum COX-2 was also decreased. Therefore, GV2 stimulation by yellow laser might enhance the recovery of spinal cord via the increase in BDNF and the decrease in inflammation, apoptosis, and oxidative stress status in the lesion spinal cord.

## 1. Introduction

Traumatic spinal cord injury, one of the most devastating neurological disorders, produces the profound negative impacts on a patient's life and socioeconomic burdens. It has been reported that the annual Asian incidence is around 12.06 to 61.6 per million [1]. This rate is varied between developing and developed countries. However, it has been estimated that the global prevalence of spinal cord injury (SCI) each year is between 250 000 and 500 000 cases [2]. Despite the high impacts on socioeconomic burdens, no effective therapeutic strategy is available.

SCI consists of 2 phases of injury including the primary and secondary injuries. Primary injury occurs as the result of the compression, contusion, stretching, or kinking of the spinal cord induced by mechanical insult [3]. Following this phase, secondary injury including inflammation, oxidative stress damage, and apoptosis occurs [4]. Currently, most of the therapeutic strategies target the secondary injury because this phase plays an important role on the hindrance of recovery process following SCI.

Recent studies have demonstrated that the stimulation of the governor vessel acupoints such as GV1, GV2, and GV6 can promote the regeneration of nerve fiber at the injury site of spinal cord [5, 6] by decreasing secondary damage via the suppression of inflammation and the stimulation of nerve growth factor release [6]. In addition to the acupuncture, laser therapy is also reported to promote axonal regrowth after spinal cord injury [7] and serves as the potential strategy for neurorehabilitation for spinal cord injury [8]. Moreover, accumulative lines of evidence have demonstrated that laser can stimulate acupoint and restore numerous neuronal deficits such as brain damage and memory [9–12]. Based on the beneficial effects of laser and acupuncture together with the capability to stimulate acupoint with laser, the improvement of functional recovery of spinal cord following traumatic injury induced by laser acupuncture has gained much attention. Several studies demonstrate that the stimulation of acupoint even at single acupoint can effectively produce the desired effects [9–12]. Therefore, we hypothesized that the stimulation at GV2 acupoint which is commonly used for treating weakness or atrophy of lower limbs by yellow laser might improve apoptosis and oxidative stress and neurological deficit in SCI rats. To elucidate this issue, this study aimed to determine the effect of laser acupuncture at GV2 acupoint on locomotor activity, oxidative stress, and apoptosis in SCI rats.

## 2. Materials and Methods

### 2.1. Animals and Experimental Protocol

Adult male Wistar rats weighed 250-300 grams were used in this experiment. All rats were purchased from National Laboratory Animal Centre, Mahidol University, Salaya, Thailand. Rats were housed in a temperature-controlled room under a 12-hour light/dark cycle and given access to food and water ad libitum. The animals were acclimatized to the laboratory to the laboratory condition for 1 week. The protocols conducted in this study were approved by the Institutional Animal Care and Use Committee Khon Kaen University, Khon Kaen, Thailand (AEMDKKU 002/2558).

All animals were randomly divided into 5 groups as follows:

Group I: Naïve intact group: all rats in this group received no treatment.

Group II: Sham operation+ sham laser acupuncture group: all rats were subjected to sham operation surgery and received no treatment

Group III: SCI + sham laser acupuncture: rats in this group were subjected to traumatic injury at T10 level

Group IV: SCI+GV2 laser acupuncture: the experimental rats in this group were induced traumatic spinal cord injury and received GV2 stimulation induced by yellow laser.

After the induction of spinal cord injury induced by traumatic injury, rats in group III-group IV were subjected to the 10-minute stimulation period by various interventions as described earlier. The stimulation was performed at 15 minutes, 6, 12, and 24 hours after spinal cord injury (SCI). After the first day, the 10-minute stimulation by various interventions were performed once daily for 7 days. The neurological deficit was assessed by using a battery test comprising of Basso, Beattie, and Bresnahan (BBB) locomotor rating scale, gross motor score. These tests were performed on days 3 and 7 after SCI. At the end of study period, histomorphology of spinal cord at the lesion level was also explored by determining the densities of survival neuron, polymorphonuclear leukocytes, Bax-positve (Bax+), caspase 3-positive (caspase3+), and Bcl-2 positive (Bcl-2+) cells in ventral horn by using histology and immunohistochemistry techniques. In addition, the activity of cyclooxygenase-2 (COX-2), interleukin-6 (IL-6), and oxidative stress status parameters including malondialdehyde (MDA) level, and the activities of superoxide dismutase (SOD), catalase (CAT), and glutathione peroxidase (GPx) were also investigated to probe for the possible underlying mechanism 24 hours after the first day of intervention.

### 2.2. Induction of Traumatic Spinal Cord Injury

After the anesthetization with Pentobarbital Sodium (50 mg/kg BW), the paravertebral muscles of experimental animals were exposed by a longitudinal incision at the midline of the back and exposed T9 to T11 vertebrae and spinal cord. The crush injury at T10 level was performed by exposing to a 15-second-extradural compression (50 g). Following this process, muscles were closed in layers, and the incision was closed by using silk sutures no.4. The surgical wound was cared with Betadine and rats were returned to their cages with the provided food and water. The rat bladders were manually voided three times a day until they were able to regain normal bladder function. Tramadol (analgesic drug) at dose of 10/kg BW and Tetracycline (antibiotics) at dose of 50 mg/kg BW were administered via subcutaneous route every 12 hours for 3 days [13].

### 2.3. GV2 Acupoint Stimulation

The stimulation of GV2, an acupoint located on the posterior midline and in the hiatus of the sacrum in prone position, was performed by yellow laser. The laser equipment used in this study was Weberneedle® Compact (Lauenförde, Germany) which can emit a wavelength of 589 nm and an output power of 50 mW and a diameter of the laser beam was 500 *μ*m. At the end of 10 minute-intervention period, the interventions were terminated. On the first day of SCI, the interventions were applied at 15 minutes, 6, 12, and 24 hours after spinal cord injury (SCI). After the first day, the 10 minute-stimulation were performed once daily for 7 days.

### 2.4. Neurological Deficit Assessments

#### 2.4.1. Locomotor Activity Evaluation (Basso, Beatie and Bresnahan, or BBB Score Test)

The locomotion, weight support and coordination capacity following SCI of animals were assessed by using the Basso, Beattie, and Bresnahan (BBB) scale [14] was evaluated. According to this method, a score of 0-21 was graded. The highest score or 21 represented complete mobility whereas 0 represented no spontaneous movement [15].

#### 2.4.2. Gross Motor Score Evaluation

In addition to BBB test, the locomotor activity was also determined by assessing motor function through movement in hindlimb and weight bearing. The locomotor impairments of rats were observed using open field grading scores ranging from 0 (no movement in hindlimb) to 10 (normal walking). The locomotor testing was carried out for 4 minutes [16].

### 2.5. Biochemical Assays

#### 2.5.1. Tissue Preparation and Protein Determination

After the last intervention, the lesion spinal tissue was removed and homogenized in 0.5 mL of ice-cold Tris-HCl buffer (50 mM, pH 7.4). Following this process, the homogenate was subjected to a 3,000 rounds per minute (rpm)-centrifugation at 4°C for 15 minutes. The supernatant was harvested and stored at -80°C until used. The protein concentration in the homogenate was determined by using a Thermo Scientific NanoDrop 2000c spectrophotometer (Thermo Fisher Scientific, USA and the optical density at the wavelength of 280 nm was determined [17].

#### 2.5.2. Determination of Oxidative Stress Status Parameters

To assess the effect of GV2 stimulation on oxidative stress status parameters, the oxidative stress status parameters, including malondialdehyde (MDA), and the activities of superoxide dismutase (SOD), catalase (CAT), and glutathione peroxidase (GPx) in the lesion spinal cord, were determined.

The level of MDA was determined by using thiobarbituric reaction [18]. In brief, an aliquot of sample tissue at the volume of 100 *μ*l was mixed with 100 *μ*l of 8.1% sodium dodecyl sulphate (SDS) (Sigma-Aldrich, USA), 375 *μ*l of 0.8% of thiobarbituric acid (TBA) (Sigma-Aldrich, USA), 375 *μ*l of 20% acetic acid (Sigma-Aldrich, USA), and 150 *μ*l of distilled water (DW). Then, the mixture was boiled in a water bath at 95°C for 60 minutes. After cooling at room temperature, 500 *μ*l of water and 2.5 ml of the mixture of n-butanol and pyridine at the ratio of 15 : 1 were added, mixed together, and centrifuged at 4,000 rpm for 10 minutes. The supernatant was harvested and determined an absorbance of 532 nm by spectrophotometer. MDA level was expressed as nmol/mg protein.

SOD activity was measured based on the ability of SOD to inhibit the reduction of cytochrome* c* by competing for the superoxide radical. In brief, 20 *μ*l of tissue sample was added to the assay mixture containing 57 mM phosphate buffer solution (KH_2_PO_4_) (Sigma-Aldrich, USA), 0.1 mM EDTA (Sigma-Aldrich, USA), 10 mM cytochrome C (Sigma-Aldrich, USA) solution and 50 *μ*M of xanthine (Sigma-Aldrich, USA) solution at the volume of 200 *μ*l. Then, 20 *μ*l of xanthine oxidase (0.90 mU/ml) (Sigma-Aldrich, USA) solution was added. The absorbance was measured at 415 nm using microplate reader. SOD enzyme (Sigma-Aldrich, USA) activities at the concentrations of 0-25 units/ml were used as standard and the results were expressed as units/mg protein [19].

Catalase activity was measured based on the rate of H_2_O_2_ disappearance. Briefly, 10 *μ*l of spinal cord homogenate was mixed with the assay mixture containing 50 *μ*l of 30 mM hydrogen peroxide ((in 50 mM phosphate buffer, pH 7.0) (BDH Chemicals Ltd, UK), 25 *μ*l of H_2_SO_4_ (Sigma-Aldrich, USA) and 150 *μ*l of 5 mM KMnO4 (Sigma-Aldrich, USA). Absorbance at 490 nm was measured using a spectrophotometer. CAT enzyme (Sigma-Aldrich, USA) at the concentration range of 0-100 units/ml was used as standard and the result was expressed as units/mg protein [20].

GPx was measured according to the procedure previously described [21]. In brief, 20 *μ*l of sample supernatant was mixed with the reaction mixture consisting of 10 *μ*l of 1 mM dithiothreitol (DTT) (Sigma-Aldrich, USA) in 6.67 mM potassium phosphate buffer (pH 7), 100 *μ*l of 1 mM sodium azide (Sigma-Aldrich, USA) in 6.67 mM potassium phosphate buffer (pH 7), 10 *μ*l of 50 mM glutathione (Sigma-Aldrich, USA) solution and 100 *μ*l of 30% hydrogen peroxide (BDH Chemicals Ltd, UK). Then, the mixture was shaken for 5 minutes before adding 10 *μ*l of DTNB (5,5-dithiobis-2-nitrobenzoic acid) (Sigma-Aldrich, USA). The absorbance at 412 nm was recorded using a spectrophotometer. The standard calibration curve was prepared by using GPx enzyme (Sigma-Aldrich, USA) at the concentration range of 0-5 units/ml. GPx activity was expressed as units/mg protein. Then, 10 *μ*l of 10 mM DTNB (5,5-dithiobis-2-nitrobenzoic acid) (Sigma-Aldrich, USA) was added and the optical density at 412 nm was recorded at 25°C over a period of 5 minutes. The standard calibration curve was prepared by using GPx enzyme (Sigma-Aldrich, USA) at the concentration range of 0-5 units/ml. GPx activity was expressed as units/mg protein.

#### 2.5.3. Assessment of Cyclooxygenase-2 (COX-2)

COX-2 activity was measured by using a commercial COX activity assay kit (Cayman Chemical, Ann Arbor, Michigan, USA). COX-2 is an enzyme that involve in inflammatory event by converting arachidonic acid to prostaglandin, an inflammatory mediator. Briefly, 10 *μ*l of tissue sample or serum, 20 *μ*l of 10 *μ*M TMPD (N,N,N′,N′-Tetramethyl-p-phenylenediamine dihydrochloride), and 20 *μ*l of 100 *μ*M arachidonic acid were transferred into 96-well microliter plates. Following a 30 minute-incubation at room temperature, the absorbance at 590 nm was recorded [22, 23].

#### 2.5.4. Assessment of Interleukin-6 (IL-6)

The level of IL-6 was determined using ELISA kit (Sigma-Aldrich, USA). The determination was performed according to the guideline protocol provided with the kit and data were expressed as pg/mg protein. In brief, an aliquot of 100 *μ*l of tissue sample or serum was added to a 96 well-plate which coated with antibody against IL-6 and incubated at room temperature for 150 minutes. At the end of incubation period, washing process was carried out. Then, the biotinylated antibody against IL-6 antibody at 100 *μ*l was added and incubated at room temperature for 60 minutes. After washing and draining the solution, HRP-Streptavidin solution at 100 *μ*l was added and incubated for 45 minutes. The washing process was carried out following the incubation. Then, an ELISA Colorimetric TMB Reagent (Item H) at volume of 100 *μ*l was added and incubated at room temperature for 30 minutes. At the end of incubation period, a stop solution (Item I) at 50 *μ*l was added and the absorbance at 450 nm was measured. Data were presented as pg/ml.

### 2.6. Histological Study

Rats were sacrificed and subjected to a transcardial perfusion with 0.9% sterile saline for 5 minutes. The spinal cord was removed and placed in 4% paraformaldehyde overnight. Following this process, the tissue was cryoprotected by incubating in 30% sucrose for 72 hours at 4°C. Serial cross sections of the lesion spinal cord were cut at 6 *μ*m thick by using a cryostat (Thermo Scientific™ HM 525 Cryostat). Then, hematoxylin and eosin (H&E) stain was performed. Briefly, the sections were stained with hematoxylin (Sigma-Aldrich, USA) 10 minutes and washed with water 1 minute (2 times). Then, the slides were immersed in 50% and 70% ethanol for 3 minutes each and stained with eosin (Sigma-Aldrich, USA) for 1 minute and dehydrated through 70, 95, 100% ethanol for 2 minutes each. The sections were cleared with xylene for 5 minutes (2 times) and mounted using DPX mountant (Merck, Germany) [24]. The photographs of sections were taken using a microscope (Axio Imager.A1, Carl Zeiss, Oberkochen, Germany) equipped with a digital camera system (Axio Cam MRc 5, Carl Zeiss). The total number of polymorphonuclear leukocyte (neutrophil) per section was counted in three randomly selected fields from the lesion site per sample at 40x magnification [25].

To assess the surviving neurons density, Nissl stain was performed. The spinal cord tissue sections were immersed in 0.2% cresyl violet (Sigma-Aldrich, USA) for 2 minutes, rinsed with double distilled water, dehydrated in ethanol solutions with increasing concentrations (70, 95, 100% 2x). Then, the sections were mounted with DPX mountant and observed under a light microscope. Normal neurons were characterized by blue staining Nissl bodies [26]. Counts were performed in three adjacent fields and expressed as the mean number of Nissl positive cells/255 *μ*m^2^.

The determination of densities of Bax-positive (Bax+), Caspase 3-positive (Caspase 3+), Bcl-2-positive (Bcl-2+), and brain derived nerve growth factor positive (BDNF+) cells were performed by using immunohistochemistry technique. In brief, spinal cord was removed and placed in 4% paraformaldehyde overnight. Then, the tissues were fixed in 30% sucrose as a cryoprotectant for 72 hour at 4°C. The spinal cord tissues were then frozen and sectioned at 20 *μ*m thick by using cryostat. The consecutive sections were collected into six-well plates containing 0.1 M phosphate buffered saline (pH 7.4) and heated with microwave (800 watt) for 10 minutes. After heating, the sections were cooled at room temperature. Then, the sections were washed in PBS 3 times, 5 minutes each. Following this process, the sections were incubated in 0.3% hydrogen peroxide for 20 minutes. After the incubation, the sections were washed in PBS 3 times, 5 minutes each again. Following this step, the sections were blocked nonspecific binding antibodies by incubating the sections in a mixture containing 0.3% Triton X-100 (Fluka Chemika, Buchs, Switzerland), 1% (w/v) bovine serum album (BSA) and 10% normal goat serum for 20 minutes at room temperature. After rinsing 3 times with PBS, the sections were incubated with one of the following primary antibodies including primary antibody against Bcl-2 (1 : 500, Abcam, Cambridge, MA, USA), Bax (1 : 500, Abcam, Cambridge, MA, USA), Caspase 3 (1 : 500; Cell Signal Technology, Danvers, MA) and BDNF (1 : 500, Sigma Aldrich, Saint Louis, USA) at 4°C overnight. After washing in PBS, primary antibodies were detected with the REAL™ EnVision™ Detection System, Peroxidase/DAB+ rabbit/mouse, (Dako, Glostrup, Denmark) by a 30 minute-incubation period for 30 minutes at room temperature. The sections were rinsed with PBS and incubated for 5 minutes with 3,3′-diaminobenzidine tetrahydrochloride (DAB) (Sigma-Aldrich, USA). Positive staining was recognized as a brown color. The control sections were stained with secondary antibody and no immunoreactive neuron was detected. The sections were mounted on gelatin-coated slides and counterstained with cresyl violet and dehydrated with graded alcohols, cleared with xylene and mounted with DPX mountant and observed under a light microscope. Immunoreactive neurons were characterized by brown granules in the cytoplasm. Counts were performed in three adjacent fields and expressed as the mean number of positive cells/255 *μ*m^2^ [27, 28].

### 2.7. Statistical Analysis

Data were expressed as mean ± SEM. The significance of differences among the groups was assessed by using one way analysis of variance (ANOVA) test followed by Tukey test. The significant change was regarded when P- value < 0.05.

## 3. Results

### 3.1. Effect of GV2 Stimulation on Neurological Deficits

Table 1 showed the effect of GV2 stimulation on BBB score of the animals on days 3 and 7 after the operation. When compared to naïve intact group, sham operation group showed no significant BBB score throughout the study period. SCI rats with sham laser stimulation at GV2 acupoint significantly decreased BBB score than sham operation group throughout the study period (P-value<.01 all; compared with sham operation group). GV2 laser acupuncture could improve BBB score at 7 days after SCI (P-value<.01; compared with SCI rats+ sham laser). The effects of GV2 laser acupuncture on gross motor score in SCI rats also showed similar pattern as BBB score as shown in Table 2. SCI rats with sham laser stimulation also showed the decreased gross motor score (P-value<.001; compared to sham operation). The stimulation at GV2 by laser significantly enhanced gross motor score (P-value<.001; compared to SCI rats+ sham laser).

### 3.2. Effects of GV2 Stimulation on Inflammation

In this study, IL-6 and COX-2 activity were used as indicators reflecting inflammation. The effects of GV2 stimulation on IL-6 and COX-2 in the lesion spinal cord were assessed and data were shown in Table 3. Sham operation failed to produce the significant change on both parameters just mentioned in both spinal cord and serum. SCI rats significantly increased both parameters (P-value<.01 and .001, respectively; compared with sham operation group) in spinal cord and serum. The stimulation of GV2 acupoint by yellow laser failed to produce significant change of IL-6 in spinal cord and serum. Interestingly, the stimulation of GV2 by yellow laser significantly attenuated the elevation COX-2 in both spinal cord and serum of SCI rats (P-value<.01 all; compared with SCI rats+sham laser group).

### 3.3. Effects of GV2 Stimulation on Oxidative Stress Status

Table 4 showed the effect of GV2 stimulation on oxidative stress parameters. It was found that sham operation produced no significant changes in MDA level and the activities of SOD, CAT, and GPx in spinal cord. SCI rats which received sham laser significantly increased MDA level but decreased SOD and GPx activities (P-value<.01; P-value<.001 and P-value<.001, respectively; compared with sham operation group). Interestingly, GV2 laser acupuncture could decrease MDA level but increased SOD activity in the lesion spinal cord (P-value<.01 and P-value<.001 respectively; compared with SCI rats+sham laser group).

### 3.4. Histological Study

**Figure 1:**
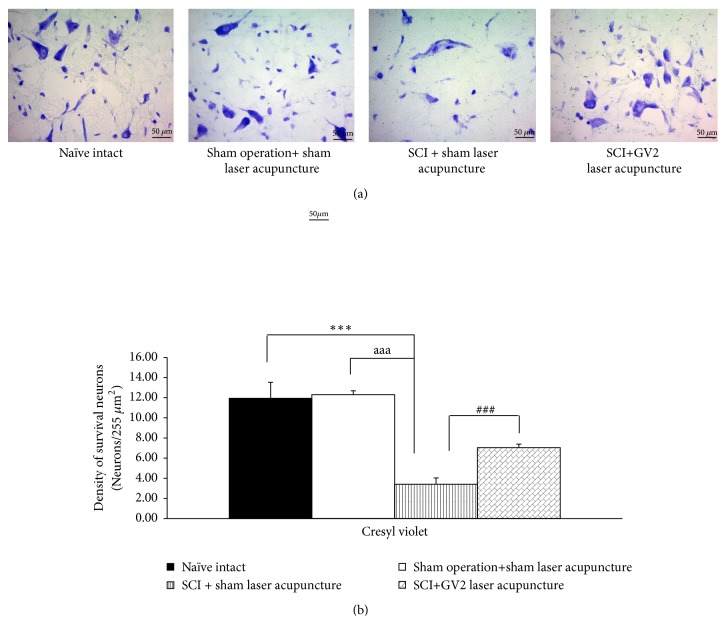
Effect of yellow laser acupuncture on survival neurons (ventral horn) in the spinal cord. Data are expressed as mean ± SEM, ^*∗∗∗*^P-value<. .001 compared with naïve intact, ^aaa^P-value<.001 compared with sham operation+ sham laser acupuncture, ^###^P-value< .001 compared with SCI + sham laser acupuncture.

**Figure 2:**
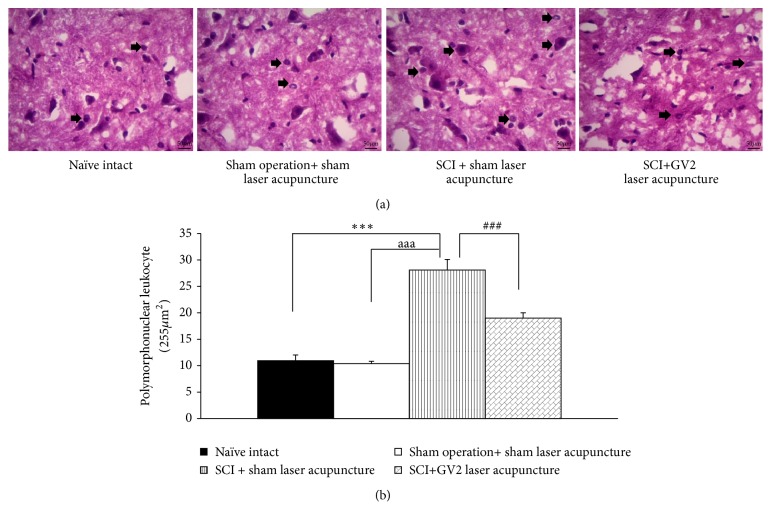
Effect of yellow laser acupuncture on polymorphonuclear leukocyte (ventral horn) in the spinal cord. Data are expressed as mean ± SEM, ^*∗∗∗*^P-value<.001 compared with naïve intact, ^aaa^P-value<.001 compared with sham operation+ sham laser acupuncture, and ^###^P-value<.001 compared with SCI + sham laser acupuncture.

Figure 1 showed the neuron density in ventral horn of spinal cord. SCI rats which received sham laser significantly decreased the density of survival neurons (P-value<.001; compared to sham operation rats). The stimulation of GV2 by laser acupuncture significantly increased neuron density in ventral horn (P-value<.001 respectively; compared with SCI rats+sham laser group). In addition, the data obtained from this study also showed that SCI rats which received sham operation significantly increased polymorphonuclear density in ventral horn of spinal cord (P-value<.001; compared to sham operation rats). This elevation was mitigated by the stimulation of GV2 acupoint by yellow laser (P-value<.001; compared with SCI rats+sham laser group) as shown in Figure 2.

**Figure 3:**
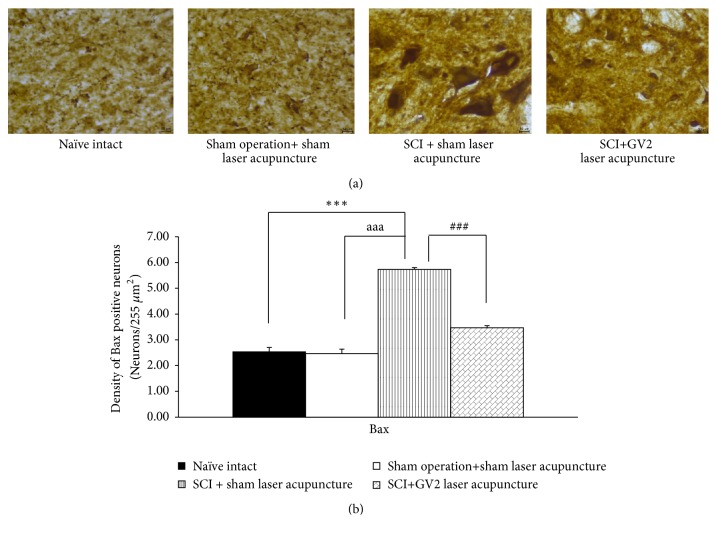
Effect of yellow laser acupuncture on Bax positive stained neurons density in ventral horn of spinal cord injury rats. Data are expressed as mean ± SEM, ^*∗∗∗*^P-value<.001 compared with Naïve intact ^aaa^P-value<.001 compared with sham operation+ sham laser acupuncture, ^###^P-value<.001 compared with SCI + sham laser acupuncture.

**Figure 4:**
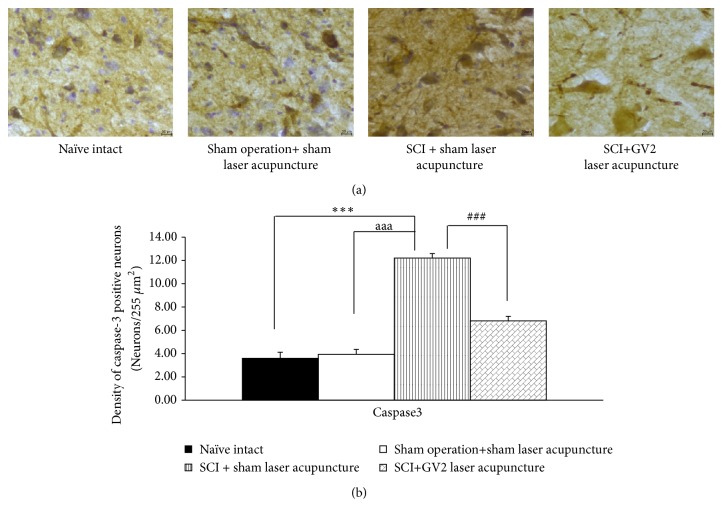
Effect of yellow laser acupuncture on Caspase 3 positive stained neurons density in ventral horn of spinal cord injury rats. Data are expressed as mean ± SEM, ^*∗∗∗*^P-value<.001 compared with naïve intact, ^aaa^P-value<0.001 compared with sham operation+ sham laser acupuncture, and^ ###^P-value<.001 compared with SCI + sham laser acupuncture.

**Figure 5:**
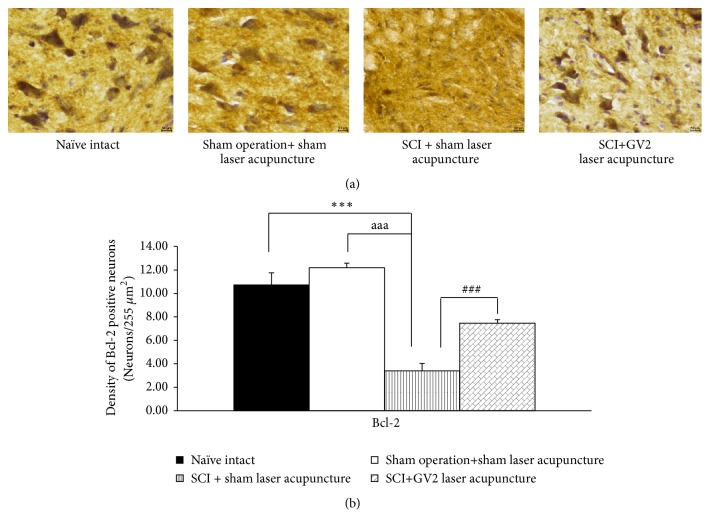
Effect of yellow laser acupuncture on Bcl-2 positive stained neurons density in ventral horn of spinal cord injury rats. Data are expressed as mean±SEM, ^*∗∗∗*^P-value<.001 compared with naïve intact, ^aaa^P-value<.001 compared with sham operation+ sham laser acupuncture, and ^###^P-value< .001, respectively; compared with SCI + sham laser acupuncture.

**Figure 6:**
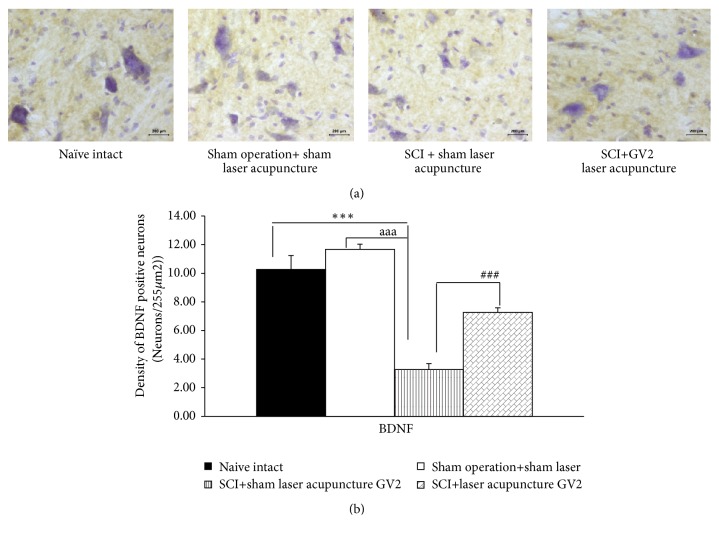
Effect of yellow laser acupuncture on BDNF-positive stained neurons density in ventral horn of spinal cord injury rats. Data are expressed as mean±SEM, ^*∗∗∗*^P-value<.001 compared with naïve intact, ^aaa^P-value<.001 compared with sham operation+ sham laser acupuncture, and ^###^P-value< .001, respectively; compared with SCI + sham laser acupuncture.

The effect of GV2 stimulation on apoptosis in ventral horn was also explored and results were shown in Figures 3–6. SCI rats which received sham laser acupuncture significantly increased Bax and Caspase 3 but decreased Bcl-2 and BDNF positive cells densities in ventral horn (P-values<.001 all; compared to sham operation). When compare to naïve control, no significant changes of all parameters just mentioned were observed in ventral horn of sham operated rats. However, the increase in Bax and Caspase 3 in ventral horn of SCI rats were attenuated by GV2 laser acupuncture (P-value<.001all; compared with sham operation group). In addition, the reduction in densities of Bcl-2 and BDNF positive cells was also mitigated by GV2 laser acupuncture (P-value<.001 all; compared with SCI rats+sham laser group).

## 4. Discussion

The current study has demonstrated that the stimulation of GV2 acupoint by yellow laser can enhance the structural lesion and functional recovery in spinal cord following traumatic cord injury. Locomotor activity of SCI rats which received GV2 laser acupuncture was improved. The decrease in COX-2, oxidative stress status, polymorphonuclear density, and apoptosis but increase in BDNF-positive cell density were also observed in SCI rats which received GV2 laser acupuncture.

It has been reported that IL-6 is the principal proinflammatory cytokine in SCI [29]. This cytokine plays roles in regulating various steps in inflammatory reactions, such as the activation and infiltration of neutrophils, monocytes, macrophages, and lymphocytes [30]. In addition, among various types of leukocytes mentioned earlier, neutrophil is regarded as the most potent triggers of post-traumatic spinal cord damage. Based on the crucial roles of both IL-6 and polymorphonuclear leukocyte (neutrophil) density, both parameters were explored in this study. Interestingly, the stimulation of GV2 acupoint by yellow laser decreased polymorphonuclear leukocyte density. Therefore, the increased survival neurons in ventral horn of spinal cord and the improved locomotor activity of SCI rats which received laser stimulation at GV2 might occur partly via the decreased inflammation and via the reduction of polymorphonuclear leukocyte density. It was found that COX-2 activity was also upregulated in SCI rats and this change was mitigated by yellow laser acupuncture at GV2 acupoint. Based on this information, we did suggest that the decrease in inflammation might also occur partly via the reduction of COX-2.

The data obtained from this study demonstrated that the SCI rats showed the reduction of the main scavenger enzymes, including SOD and GPx, leading to the excess of superoxide anion and hydrogen peroxide and the attack of polyunsaturated fatty acid (PUFA) in the membranes of various organelles resulting in the elevation of malondialdehyde (MDA) level. These results were in agreement many the previous studies [31, 32]. GV2 stimulation by yellow laser could attenuate the reduction of SOD and the elevation of MDA level of SCI rats. In addition, it has been reported that oxidative stress can induce inflammatory response which in turn leads to apoptosis and neurological deficit [33]. Therefore, the decreased inflammation in SCI rats might occur not only via the reduction of polymorphonuclear leukocyte density but also via the reduction of oxidative stress. However, the reduction of inflammation can also decrease oxidative stress [34]. Therefore, the reduction of oxidative stress induced by the stimulation induced by the stimulation of GV2 also occurs partly due to the reduction of inflammation. Interestingly, the stimulation of GV2 by yellow laser could enhance SOD activity. It has been reported that SOD, an important antioxidant enzyme, catalyzes the dismutation of superoxide anion (O_2_^−^) to H_2_O_2_ [35]. Therefore the reduction MDA observed in SCI rats which received GV2 stimulation by yellow laser might occur partly via the increase in SOD which in turn decreased H_2_O_2_ and the attack of free radicals to lipid component of membrane leading to the reduction of neurodegeneration.

Following SCI, apoptosis also occurs and plays a role on the functional disability [36, 37]. This process is regulated by Bcl-2, Caspase 3, and Bax [38, 39]. Our data revealed that following SCI, Bax and Caspase 3 positive cells were upregulated whereas Bcl-2 positive cell was downregulated. These data were in agreement with the previous study [39]. Interestingly, our data demonstrated that the stimulation at GV2 acupoint significantly increased the density of Bcl-2 positive cell but decreased the densities of Bax and Caspase-3 positive cells in ventral horn of the lesion spinal cord.

The current data showed that the density of survival neurons in ventral horn of spinal cord increased. The underlying mechanism is most likely to be associated with the decreased apoptosis (density of Bax-positive cell decreased but density of Bcl-2-positive cell increased). Therefore, the growth factor may contribute a role on this process. However, the growth factor which contributes essential role on the recovery of spinal cord following injury and has gained much attention as therapeutic strategy against spinal cord injury is brain derived growth factor (BDNF) [40]. It can rescue neurons from degenerative atrophy and apoptotic cell death [41, 42]. The current results showed that laser acupuncture at GV2 acupoint significantly enhanced BDNF positive cell in SCI rats. Therefore, the increased survival neuron density together with the decreased apoptosis might be associated with the increase in BDNF which in turn enhanced the survival of neuron and suppressed apoptosis.

Taking all data together, our data suggested that SCI rats which received GV2 stimulation by yellow laser significantly enhanced BDNF producing cells which in turn increased the survival neuron and suppressed apoptosis of neurons in ventral horn of spinal cord. In addition GV2 laser acupuncture also mitigated oxidative stress status and inflammation. These processes also play the roles on the increase neuron density in ventral horn which in turn improved locomotor activity of SCI rats. The elevation of glial cell derived growth factor (GDNF) which plays an important role on the promotion of axonal regeneration and myelination may also involve the improvement of functional outcome of spinal cord following traumatic injury. However, this required further studies.

## 5. Conclusion

In conclusion, the stimulation at GV2 acupoint by yellow laser is the potential novel intervention to improve both motor deficit and neurodegeneration after traumatic injury in ventral horn of spinal cord. It should provide health benefit for many disability patients and decrease annual healthcare budget related to the management of disability of traumatic spinal cord injury. However, the clinical trial study is still essential before moving forward for further application.

## Figures and Tables

**Table 1:** Effect of yellow laser acupuncture on the BBB score in rats induced spinal cord injury.

**Treatment groups**	**Baseline**	**Day 3**	**Day 7**
Naïve intact	21.00±0.000	21.00±0.000	21.00±0.000
Sham operation+ sham laser acupuncture	21.00±0.000	21.00±0.000	21.00±0.000
SCI + sham laser acupuncture	21.00±0.000	0.00±0.000∗∗∗,aaa	0.00±0.000∗∗,aa
SCI+GV2 laser acupuncture	21.00±0.000	0.67±0.333	9.17±0.167###

Data are expressed as mean±SEM and ^*∗∗*, *∗∗∗*^P-value<.01 and .001, respectively; compared with naïve intact, ^aa, aaa^P-value<.01 and .001, respectively; compared with sham operation+ sham laser acupuncture, ^###^P-value< .001, respectively; compared with SCI + sham laser acupuncture.

**Table 2:** Effect of yellow laser acupuncture on the gross motor score in rats induced spinal cord injury.

**Group**	**Baseline**	**Day 3**	**Day 7**
Naïve intact	10.00±0.000	10.00±0.000	10.00±0.000
Sham operation+ sham laser acupuncture	10.00±0.000	10.00±0.000	10.00±0.000
SCI + sham laser acupuncture	10.00±0.000	0.00±0.000^*∗∗∗*,aaa^	0.00±0.000^*∗∗∗*,aaa^
SCI+GV2 laser acupuncture	10.00±0.000	1.00±0.447	3.67±0.61^###^

Data are expressed as mean±SEM, ^*∗∗∗*^P-value<.001 compared with naïve intact, ^aaa^P-value<.001 compared with sham operation+ sham laser acupuncture, and ^###^P-value<.001 compared with SCI + sham laser acupuncture.

**Table 3:** Effect of laser acupuncture at GV2 on the alterations of cyclooxygenase-2 (COX-2) and interleukin-6 (IL-6) levels in spinal cord and serum.

Treatment	Spinal cord		Serum	
	COX-2 (ng/dL)	IL-6 (pg/mL)	COX-2 (ng/dL)	IL-6 (pg/mL)
Naïve intact	17.94±0.54	0.41±0.08	17.24±0.89	0.05±0.31
Sham operation +sham acupuncture	18.75±1.53	0.41±0.11	18.59±1.63	0.05±0.02
SCI+sham acupuncture	28.64±1.50^*∗∗∗*,aaa^	1.60±0.60^*∗∗*,aa^	27.40±1.35^*∗∗∗*,aaa^	0.21±0.01^*∗∗*,aa^
SCI+laser acupuncture GV2	19.96±1.25^##^	0.65±0.16	19.91±1.20^##^	0.11±0.01

Data are expressed as mean ± SEM, ^*∗∗*, *∗∗∗*^P-value<.01 and .001 respectively; compared with naïve intact, ^aa, aaa^P-value< .01 and .001, respectively; compared with sham operation+ sham laser acupuncture, ^##^P-value<.01; compared with SCI + sham laser acupuncture.

**Table 4:** The effect of yellow laser acupuncture on oxidative stress parameters in spinal cord.

**Group**	**MDA level (nmol/mg protein)**	**SOD activity (U/mg. Protein)**	**CAT activity (U/mg protein)**	**GPx activity (U/mg protein)**
Naïve intact	0.07±0.006^##^	27.13±1.434^###^	49.37±5.691^###^	5.14±0.753^###^
Sham operation+ sham laser acupuncture	0.07±0.009^##^	25.37±1.797^###^	46.16±10.049	4.91±0.658^###^
SCI + sham laser acupuncture	0.13±0.014^*∗∗*,aa^	10.90±1.039^*∗∗∗*,aaa^	13.47±0.992^*∗∗∗*^	1.54±0.114^*∗∗∗*,aaa^
SCI+GV2 laser acupuncture	0.08±0.003^##^	19.70±1.062^*∗∗*,###^	32.01±1.047^*∗∗*^	3.39±0.091

Data are expressed as mean ± SEM, ^*∗∗*, *∗∗∗*^P-value<.01 and .001 respectively; compared with naïve intact, ^aa, aaa^P-value<.01and.001 respectively; compared with sham operation+ sham laser acupuncture, ^##, ###^P-value<.01 and .001, respectively, compared with SCI + sham laser acupuncture.

## Data Availability

The data used to support the findings of this study are available from the corresponding author upon request.
